# *You just have to have other models, our DNA is different:* the experiences of indigenous people who use illicit drugs and/or alcohol accessing substance use treatment

**DOI:** 10.1186/s12954-020-00366-3

**Published:** 2020-03-24

**Authors:** Jennifer Lavalley, Shelda Kastor, Malcolm Tourangeau, Western Aboriginal Harm Reduction Society, Ashley Goodman, Thomas Kerr

**Affiliations:** 1 British Columbia Centre on Substance Use, 400-1045 Howe Street, Vancouver, British Columbia V6Z 2A9 Canada; 2 Western Aboriginal Harm Reduction Society, Vancouver, British Columbia Canada; 3 Department of Medicine, University of British Columbia, St. Paul’s Hospital, 608-1081 Burrard Street, Vancouver, British Columbia V6Z 1Y6 Canada

**Keywords:** Indigenous peoples, Community-based participatory research, Decolonizing, Indigenous methodologies, Marginalized populations, Substance use treatment

## Abstract

**Objectives:**

In Canada, and elsewhere, indigenous peoples who use illicit drugs and/or alcohol (IPWUID/A) commonly experience vulnerability and a disproportionate burden of harm related to substance use. In Vancouver, Canada, there are concerns that inequitable access, retention, and post treatment care within substance use treatment programs may exacerbate these harms. This study sought to understand the policies and practices with the potential to produce inequities and vulnerabilities for IPWUID/A in substance use treatment, situate the vulnerabilities of IPWUID/A in substance use treatment within the context of wider structural vulnerability of IPWUID/A, and generate recommendations for culturally safe treatment options.

**Methods:**

This research employed a qualitative indigenous-led community-based approach using the indigenous methodology of talking circles to explore experiences with substance use treatment. Under the participatory research framework, community researchers led the study design, data collection, and analysis. Talking circles elicited peers’ experiences of substance use treatment and were audio-recorded and transcribed.

**Results:**

The talking circles identified three key themes that illustrated the experiences of IPWUID/A when accessing substance use treatment: (a) barriers to accessing detox and substance use treatment; (b) incompatible and culturally inappropriate structure, policies, and procedures within treatment programs, such as forced Christianity within treatment settings; and (c) the importance of culturally relevant, peer-led substance use treatment programming.

**Discussion:**

Our work demonstrates that some IPWUID/A have limited access to or retention in mainstream treatment due to excessive waiting times, strict rules, and lack of cultural appropriate care while in treatment. However, IPWUID/A narratives revealed strategies that can improve IPWUID/A access and experiences, including those informed by the diverse perspectives of IPWUID/A and those that include trauma-informed and culturally safe practices.

## Introduction

In Canada, substantial disparities in health and healthcare access persist among those who are structurally vulnerable and marginalized (i.e., people with low income, the homeless, and racialized minorities) [1]. Among those most affected by health disparities in Canada are indigenous peoples, namely First Nations, Inuit, and Métis people [2–5]. These health disparities have been associated with social, economic, cultural, and political inequities; all of which have caused considerable health-related and social harms for Indigenous peoples in Canada [4], such as lower life expectancies, higher rates of incarceration, poverty, and chronic health conditions [6]. Importantly, indigenous health cannot be understood outside the context of colonization [2]. Healthcare inequalities among indigenous peoples are perpetuated by systemic racism and are intrinsically linked to the ongoing structural and systemic inequalities that result directly from the legacy of colonization [2, 7, 8]. It has been well documented that colonization is a key determinant of health for indigenous peoples [2, 7, 8]. Key social determinants of health—including education, employment, income, and gender [5]—have shaped a wide range of health capacities and vulnerabilities for indigenous peoples [3].

It is widely recognized that indigenous peoples in Canada experience inequitable access to healthcare as well as a disproportionate burden of harm related to substance use. In 2017, indigenous peoples who use illicit drugs and/or alcohol (IPWUID/A) accounted for 14% of overdoses [9] in British Columbia (BC), Canada, despite only representing 5.9% of BC’s population [10] and just 2.6% of the population in Canada [11]. Unfortunately, while access to healthcare services is largely regarded as an important determinant of health [12–14], past research has revealed that access to healthcare is too often limited for racialized minority drug users [2, 12]. Further, stigma and discrimination compound the negative health impacts related to substance use [14]. While IPWUID/A carry a disproportionate burden of health-related problems associated with drug use, the experiences of IPWUID/A in substance use care have not been well characterized.

A prior study has documented the impacts of systemic racism within the healthcare system and how these social-structural forces shape social norms and behaviors of healthcare providers when treating IPWUID/A, consequently influencing the healthcare experiences of IPWUID/A [13]. Utilizing a structural approach helps to contextualize the ways the historical structure of colonialism—enacted through colonial policies such as the Indian Act (a statute that provides the federal government of Canada control over who is defined as an “Indian” and the enforcement of the on-reserve band system)—influences contemporary indigenous health inequities [2].

The need to understand the experiences of indigenous peoples accessing substance use treatment is particularly important in Vancouver, Canada’s major western urban center—home to the largest urban indigenous population in BC [15], most acutely visible in Vancouver’s Downtown Eastside (DTES) [16]. The DTES is a low-income neighborhood that is a site of Canada’s largest street-based illicit drug scene, characterized by high rates of poverty, substance use, violence, and homelessness [17, 18]. Despite the well-documented health disparities among IPWUID/A, research exploring the distinct experiences of IPWUID/A when accessing substance use treatment is limited. To address this gap, this study seeks to understand how intersecting forms of discrimination and oppression shape the experiences of IPWUID/A when accessing substance use treatment.

### Current project

This research was undertaken in Vancouver, British Columbia. Presently, there are approximately 14, 000 indigenous peoples in Vancouver, which account for 2.2% of the population [19]. Community members involved in this research consisted of individuals who identify as IPWUID/A living in the inner-city neighborhood of Vancouver’s Downtown Eastside (DTES). The DTES is characterized by high rates of poverty, homelessness, substance use, mental health issues, and violence, along with vast social and economic marginalization [17, 18, 20–23]. The DTES is also distinctive in that at least one third of the city’s total indigenous population live in or near the area [24], and it is known for its “open” drug scene [25].

The Western Aboriginal Harm Reduction Society (WAHRS) and academic researchers from the BC Centre on Substance Use (BCCSU) comprised the research team for this project, which included a designated indigenous research coordinator. WAHRS is an indigenous-led organization, under the umbrella of Vancouver Area Network of Drug Users (VANDU) (a peer-based organization), who represent current or previous users of illicit drug and or illicit alcohol dedicated to harm reduction and the improvement of the quality of life of other IPWUID/A by encouraging the development of support, education, and training programs that reflect the values of indigenous peoples. WAHRS Board Members were both community researchers and participants within the context of this project and selected both the research methodology and research topic of interest to the community and were responsible for data collection and analysis. The BCCSU provided academic and research support.

The BCCSU has a long-standing research relationship with the DTES community. The BCCSU partnered with WAHRS to conduct community-based research to explore their research experiences. For this project, WAHRS Board Members had active roles as community researchers and employed indigenous ways of knowing and sharing through the project.

The research team explored and shared their personal experiences with substance use treatment among their peers through the use of talking circles. This article is designed to elucidate how indigenous peoples who are faced with multiple health-related disparities experience mainstream addiction treatment programs.

## Methods

The methods used in this study have been previously described [13, 26]. The research presented in this article draws on a series of peer-facilitated talking circles exploring indigenous community members’ experiences with substance use treatment. Talking circles were selected as a culturally appropriate research method that utilizes an indigenous framework—grounded in indigenous knowledge and reflects the values and beliefs of indigenous communities [27, 28]. Indigenous ways of knowing—or culturally specific ways of creating knowledge in community practices [28], have been embedded throughout our entire research design. Indigenous-based methods prioritize indigenous knowledge, capacity building, and community healing within the research process itself [27, 28], understanding that indigenous peoples’ experiential knowledge of harm reduction models is rooted in their specific socio-cultural contexts which, in Canada, are shaped by colonialism. Further, talking circles were facilitated by two WAHRS Board Members (community researchers) and participation was open to the WAHRS membership. Participation by individuals was limited to one talking circle. Using convenience-sampling, talking circle participants were recruited during the community organization’s weekly membership meetings through a draw-system, which was limited to ten participants. While serving as facilitators, WAHRS Board Members also contributed their own lived experiences during the talking circles. In total, three talking circles were completed, averaging 1 h in length. Ethical approval was granted by the Providence Healthcare/University of British Columbia Research Ethics Board.

Each talking circle involved introducing the research topic to the group by the facilitators who then initiated the talking circle by sharing their personal experiences with addiction treatment as an IPWUID/A living in the DTES. Participants took turns speaking while holding an eagle feather, a chosen cultural symbol signifying honor and respect. If desired, participants were allowed to pass on speaking. Talking circles were audio-recorded and transcribed verbatim and reviewed for accuracy and preliminary themes by the community researchers and research coordinator.

Most of the Board Members participated in the research as community researchers. Qualitative analysis of the data was undertaken individually and as a group and involved a detailed review of the transcripts. Preliminary themes and descriptive quotes emerged and were presented to the WAHRS Board for review. The research team read each transcript aloud and then coded line-by-line by hand to identify key themes and recommendations. WAHRS presented a summary of the analysis and findings to a large membership meeting, which was attended by 35 members. Members were encouraged to provide insight on the research topic and findings and also to contribute to the recommendations.

Following data analysis, data was broadly reviewed by an indigenous researcher from the BCCSU for the purpose of drafting an academic peer-reviewed research article. Several joint meetings were held with WAHRS Board Members to share, discuss, and revise the manuscript. Manuscript meetings were audio-recorded and transcribed verbatim by the first author. The first author reviewed the transcripts to ensure the community researchers’ voices were accurately represented in the writing of this manuscript.

## Results

The following key themes emerged from the stories of IPWUID/A and were chosen to best represent and emphasize IPWUID/A experiences with accessing substance use treatment. Participants’ ages ranged from 19 to 70. Participants all self-identified as indigenous (*n* = 35), with all also identifying as cisgender; men (*n* = 27), women (*n* = 8). The talking circles illustrated high rates of negative experiences and dissatisfaction with accessing substance use treatment programs and points to a lack of culturally safe and appropriate care. Participants consistently expressed dissatisfaction towards barriers when trying to access treatment, highlighting extensive wait-times, and incompatible and culturally inappropriate structure, policies, and procedures within treatment facilities, including in-patient and detox services. Participants also emphasized the importance of culturally relevant treatment programs and supports in the context of “recovery” and sought to draw attention to what they consider to be most effective, which included culturally relevant, peer-led programming.

### “I lost faith getting into detox”: access to detox and treatment services and aftercare

Overarching themes evident in participants’ stories were limited access to substance use care facilities and the lack of post treatment support. This observed lack of access and support was framed within a variety of experiences, including the extensive duration of wait times, the structure and rules within facilities, the lack of support post treatment, and the absence of accommodation for the realities of indigenous people.

Accounts regarding waiting times and admission to substance use treatment were fairly consistent. Many participants referenced long wait times at treatment facilities that prevented them from accessing services and support in relation to substance use. Waiting times were commonly influenced by both individual and systemic factors that influenced how and/or if participants received treatment. For example, one participant shared his experience about not being able to access detox as an illicit drinker due to long wait times:

Lost faith getting into detox. I found when I go to phone in they tell me to call back so I call [the] next day and they tell me to call back again and again and again. We all know they’ve got beds but just testing us. But by [the] time they accept me then it’s too late. I don’t have patience and so I am an illicit drinker. (Male participant #9, Talking Circle #5)

Participants drew upon a seemingly collective narrative about the difficulties of accessing substance use care. Many participants noted that they had made efforts to attend detox and/or treatment services. However, while it was noted that various services and supports were in place locally, when it came to accessing substance use treatment, a range of barriers reduced their chances of receiving the treatment they sought.

What I’ve noticed about Vancouver it’s difficult to get into treatment. Don’t want to be controversial, but seems [there’s] always a free meal, free shelter, clothing, bus tickets, it seems enabling. They make it hard to get into treatment. [I’ve] wanted to be clean [for] 6 months and it’s an impossibility. That’s what I’ve observed down here. I understand places like Insite [local supervised injection site] and don’t want to use words [like] enable, but I wish they would make them available to me. (Male participant #10, Talking Circle #6).

Further, participants’ accounts of the waiting period often centered around treatment facilities not being sensitive to the “fleeting” moments where people felt ready to attend. As a participant who attended treatment on several occasions shared, *“*By the time they tell you to phone back, you don’t want to go in there.” The following excerpt illuminates the harmful consequences of wait times, which ultimately influenced this participant’s decision not to attend detox.

My experience going through detox. I don’t understand why they don’t have [it] so you can actually get in! Drink ruby [i.e., rubbing alcohol]; drink List’ [i.e., mouthwash]; drink sherry; rice wine, but there’s times when people want to clean up. I don’t find it’s helpful here though, it’s harmful. If I go cold turkey I’ll go to seizures and die. What’s the point? By the time they tell you to phone back you don’t want to go in there. (Male participant #9, Talking Circle #6)

Participants also generally reported low levels of confidence in aftercare engagement and support from substance use treatment programs. This same participant further elaborated on the lack of sustained recovery post treatment as follows:

I spent six months in treatment center. Six months clean, but if you don’t have a plan you go back to the street. You go back where you started. You have to have someone or something to guide you. Don’t want to talk too much, but you really do need a body to be with you through it. (Male participant #9, Talking Circle #6).

### “Residential treatment is other people’s rules”: structure and rules within substance use treatment programs

Some participants expressed that they were expected to abide by others’ rules and that these expectations were a factor on whether or not they were successful in attending and/or completing substance use treatment. These expectations served to reinforce indigenous peoples’ marginal status within substance use treatment models, while also privileging non-indigenous peoples. For example, one man reported getting discouraged by abiding by “other people’s rules”:

Residential treatment is other peoples’ rules. I get discouraged easy. Detox not much to do. Not a lot of options out there. (Male participant #3, Talking Circle #6).

Another participant expressed similar frustrations with the structure and rules throughout her time at a treatment center.

They tell me everything, gets so overwhelming and want to go, but the one lady showed me a schedule 7am-10pm; everything was scheduled. I don’t want people to tell me what to do. (Female participant #1, Talking Circle #6).

For other participants, they simply did not believe in the effectiveness of treatment. Here, participants described their struggle to be “successful” within the context of mainstream substance use treatment programs. For example, a female participant stated, “I don’t believe in their system. I’ve tried 6 or 7 times and I try and try but keep failing them and their system.” (Female participant #2, Talking Circle #5). Several “failed” attempts at completing treatment produced a perception for participants in relation to the outcomes and effectiveness of treatment. Another participant also noted, “I’m sick and addiction centers don’t work for me”. In these instances, participants’ stories revealed the ways in which their experiences attending in-patient treatment and/or detox centers did not produce satisfactory outcomes, therefore exacerbating the marginalization of indigenous peoples from mainstream treatment models and interventions.

### “Christian-based recovery doesn’t flow. You just have to have other models. Our DNA is different”: what is not working

Participants frequently positioned substance use treatments as spaces that did not accommodate their needs—that is, settings that did not recognize or validate the unique lived experiences of indigenous peoples. The operational modes of mainstream substance use treatment models decreased access among indigenous people by not being accommodating of their unique lived experiences and realities, which includes disregarding of the complex experiences of intergenerational trauma rooted in colonialism, the lack of cultural appropriate practices, and the prioritization of Christian-based models. Participants routinely described that treatment centers did not take into account their experiences of trauma. This was particularly problematic as most participants shared traumatic stories that intensified their experiences with substance use. As one participant stated, “They [substance use treatment programs] need more counselling. Need to know why people are using”. Similarly, a female participant who attended in-patient treatment shared:

Wish I could find a treatment center who could understand my past and understand me. Last time [I] didn’t have a counsellor. I left with all this stuff on my shoulders. I let it out on the wrong people. Only thing could do is sit down and cry (Female participant #9, Talking Circle #4)

Another participant noted that, “Treatment is for helping me to dig deep in me to get issues taken care of, but they don’t do that.” For most, the value of acknowledging and understanding one’s experiences of trauma was tied to participants’ feelings around the effectiveness of treatment and whether or not they wanted to attend in the first place.

Moreover, although ideologically driven substance use treatment models (e.g., faith-based models) may occupy an important niche for some, we documented that Christian-based models heightened participants’ vulnerability to feeling unsafe. One participant shared her opinion on Christian-based programs stemming from her experience with Alcoholics Anonymous (A.A.). She stated, “A.A. doesn’t help! It triggers me to want to drink. Christian programs make me want to kill myself. I grew up in that and thought I was going to hell”. (Female participant #1, Talking Circle #6). Another participant expressed similar concerns for indigenous people having no option other than to partake in “Christian-based recovery”: “Christian-based recovery doesn’t flow. You just have to have other models. Our DNA is different” (Male participant #5, Talking Circle #6).

Stories of trauma were also consistently attached to people’s experiences surrounding family breakdown. The historical roots of intergenerational trauma were evident in many of the participants’ stories and exposed a central tension of mainstream substance use treatment programs in relation to indigenous peoples. Most inpatient treatment programs did not allow parents to bring their children with. Participants reported that, because of their role as caregivers, attending treatment jeopardized their ability to keep their children in their care. Accessing substance use treatment became, at times, the only way people were allowed to keep their children in their care. For example, a female participant explained being forced into treatment in order to keep her children: “I couldn’t keep my children without going to treatment, so they’d take me and I felt lost because they took [my] kids. My crutch is from my broken heart from being torn apart from my kids” (Female participant #2, Talking Circle #5).

At times, participants had to choose between keeping their children and attending treatment. A female participant described how she had to choose between taking care of her granddaughter or attending treatment:

I went with my granddaughter. She was talking to me about going to treatment with me. But daughter and social worker didn’t want her to go with me. She said that my life would be different if they allowed me to go. I think it’s important for me to go with my granddaughter. I would if I could have. (Female participant #1, Talking Circle #5).

In these stories, one can observe broader themes of colonization and the subsequent intergenerational impacts, including the overrepresentation of indigenous children in the child welfare system. Here, a female participant describes her experience as a child in foster care:

You don’t know what childhood is. In [my] first foster home, you know, 3 years old, it was terrible. I remember the rejection. You get taken off in a plane. What is this? We were just little children. Who are these people? (Female participant #8, Talking Circle #4)

Further, trauma from her own experiences in foster care as a child rendered her vulnerable to having her children apprehended.

Finally I had children and the welfare said I had to do treatment or they’d take my kids away. So I said ok, send me a ticket. I went to treatment; was clean for three years. The I said I could celebrate one time and they took my kids. Someone ratted on me. I was in PG [city name] then. I had to come down here. (Female participant #8, Talking Circle #4)

The last excerpt seamlessly reflects the ways in which intergenerational trauma is perpetuated through contemporary colonial policies and practices (i.e., child welfare system): “The welfare stole me from my family and they stole my children and there is a generation gap that needs to be healed. I pray to the Creator to bring me this healing” (Female participant #2, Talking Circle #5).

Despite efforts to access support for substance use to improve well-being, participants were often confronted with policies (i.e., policies that prevent people from bringing their families to treatment) that created exceptional barriers to receiving support. Such policies compromised participants’ agency to make decisions on their own health.

### The importance of culture and peer support: what does work?

Participant’s discussions of “what works” in the context of substance use care were most often tied to trauma-informed and culturally relevant practices, keeping families together, and peer support. As previously noted, participants stated that they were often expected to abide by Christian-based models within substance use treatment programs and that these expectations reinforced trauma related to past experiences of forced Christianity. Participant prioritization of culturally relevant practices demonstrates the extent to which safety within substance use treatment programs is intrinsically connected to access to culture. Most raised concerns about the lack of indigenous-focused cultural practices and support within treatment centers. For most, these were central components that facilitated safety, wellness, and care for indigenous peoples within treatment programs. Indigenous models of health care—including substance use care—were discussed in ways that consider the health of the whole communities and embodied specific conceptualizations of health and wellness. One woman described her dream as follows:

I had a dream. Called a healing ranch. I dreamed I had a longhouse. People can come and me and my daughters and granddaughters will greet people who come for hard times and we will say welcome. We would have horses and geese and natural foods. (Female participant #2, Talking Circle #5).

She further elaborated on her ideal “treatment center” as a place that centered on indigenous teachings and ways of healing, describing the engagement in cultural practices, such as “building cabins, carving, beading, and praying to the Creator.” Two other participants described the need for “holistic” treatment, emphasizing the need to participate in cultural practices such as “smudging” and “sweat-lodges” as part of recovery.

Moreover, several participants emphasized the significant impact of peer influence and support with respect to receiving substance use care. Notably, participants characterized peer support as a connection to peer-based substance use programs, such as drug user organizations like VANDU, which works to improve the lives of people who use drugs through user-based peer support and education. Receiving “treatment” was often framed within the context of participants having agency over how they perceived “treatment.” As one participant explained:

I’ve never been in treatment center but tried to get into detox. My doctor got me into Onsite. VANDU is my treatment. [I] haven’t used down for five years. This place keeps me straight and alive. (Female participant #8, Talking Circle #5)

Participants did not necessarily view “getting clean” or attending mainstream substance use treatment as an indicator of being “in recovery,” rather participants, at times, would solely view peer support and harm reduction practices as their recovery. Below are two examples that demonstrate how participants highly valued peer-supported harm reduction. One participant said, “I take VANDU as my treatment center” (Male participant #4, Talking Circle #5). Another noted, “I’ve gotta thank VANDU because a day at VANDU is another day clean for me and I’m going to stick with that treatment.” (Male participant #10, Talking Circle # 5). Additionally, an indigenous woman discussed the importance of having indigenous-led peer support within such organizations: “I have been fighting with addiction and talking to my counsellor at [health clinic], but no offense, I just need a Native woman who’s been where I’ve been” (Female participant #3, Talking Circle #6).

## Discussion

In summary, participants characterized mainstream substance use treatment as futile environments for “recovery” that not only invalidated their lived experienced as indigenous peoples, but also forced them to engage within a setting framed by incompatible values. Participants’ accounts most underscored how lengthy waiting times discouraged them from accessing substance use treatment. Highly structured and Christian-based treatment models further undermined their lived experiences as indigenous peoples and usually did not account for their experiences of trauma. However, their stories revealed hopeful frameworks and interventions that can strengthen IPWUID/A engagement and retention in substance use treatment, including culturally rooted and trauma-informed interventions that accommodate the lived experiences of indigenous peoples, including peer-led support and harm reduction.

Consistent with past research undertaken in other colonized countries (i.e., Australia, New Zealand, USA), our findings may extend to other colonized settings where the need for culturally appropriate treatment models are needed [29–31]. Our findings expand upon previous research on the ongoing impacts of colonialization and how these collective experiences continue to shape the everyday realities and health outcomes of indigenous peoples [2, 5]. Therefore, the ongoing impacts of colonization cannot be separated from any discussion of indigenous peoples’ experiences and, in this case, is central to the discussion surrounding IPWUID/A experience accessing mainstream substance use treatment. Critical to this conversation is an understanding of the historical, political, and social determinants of health. Researchers have linked the intergenerational impacts of colonization, including but not limited to the residential school experience, ongoing colonial policies (i.e., the Indian Act) and the Sixties Scoops, but also to a number of social and health inequities experienced by indigenous people [2, 3, 13], such as significantly higher morbidity and mortality rates, and limited access to healthcare services [2, 3, 32].

Our findings underscore that participants viewed lengthy wait times as a barrier to accessing substance use treatment. Long waiting lists and treatment linkage have been identified as a common barrier to utilizing drug and alcohol treatment [33–35]. In many cases, participants described their desire to attend treatment as “fleeting”—in that their desire to connect and engage with treatment services diminished during lengthy waiting periods. These findings demonstrate that excessive waiting periods dissuaded participants from receiving support to improve their health and well-being, as the need to receive services for substance use is often urgent. Building on findings regarding the factors associated with treatment retention [36], we found that structural settings (i.e., operational policies and procedures within treatment facilities) reduce treatment access and retention among IPWUID/A.

It is worth mentioning that the distinctions between faith-based and “traditional” and/or secular treatment models remain rather unclear, given the world-wide acceptance of a 12-step philosophy, which emphasizes spirituality and “higher power” [37]. Therefore, it is challenging to determine an operational definition of faith or religious-based programs. However, participants’ accounts spoke to the Christian undertones that permeated most mainstream substance use treatment programs. These connections reveal how inextricably linked mainstream “traditional” and faith-based treatment models are. Participants commonly described their negative perceptions of and experiences with Christian or faith-based recovery substance treatment programs. As discussed in the “Introduction” section, the intergenerational impacts of colonialism have fundamentally shaped the everyday realities of indigenous people [2, 38] in Canada and elsewhere. Christianity and colonialism are closely linked since one of the major tools of colonialism has been to assimilate and convert indigenous peoples into Christianity [39], and for some, Christian-based treatment reiterated the ongoing oppression and structural violence that indigenous peoples continue to experience, leaving participants to re-live these experiences of trauma.

It is crucial to acknowledge the significant impact that colonization and colonial policies have had on the social and health disparities experienced by indigenous peoples in Canada and elsewhere [1–8]. Research suggests that unstable housing, forced displacement, and relocation to reserves, forced removal of children through child welfare systems and residential schools, and limited access to healthcare services have unquestionably affected the health and well-being of many indigenous peoples [1–8]. As such, given the harmful impact that colonization has had on indigenous peoples’ lives and wellness, our findings lend further support to why efforts to address the health inequities among indigenous peoples should involve understanding the contextual determinants of health and broader systems of oppression.

Trauma-informed care means accommodating the needs of trauma survivors, recognizing their vulnerabilities and strengths, and providing survivors with the support that ensures they have autonomy and power in their healing [40, 41]. Greater efforts to develop or adapt interventions addressing intergenerational trauma that not only addresses the needs of IPWUID/A in treatment, but recognizes the complex relationship between trauma and systematic oppression are needed. Among indigenous women, these experiences of trauma were compounded by the lack of accommodation for children within treatment centers, despite evidence of the increased retention for women who kept, had childcare alternatives, and/or were allowed to take their children with them to residential treatment [40, 41]. The significance of delivering community-based treatment that provides support to women and their families is consistent with previous work that identified women’s success in treatment [42, 43]. Peardonville House, located in Abbotsford, BC, offers women the opportunity to bring under-school-age children with them to treatment and may be a model to look towards.

The striking overrepresentation of indigenous children in today’s child welfare system in Canada, and elsewhere, directly resulting from the residential school experience, the Sixties Scoop, and other tools of colonization (i.e., the Indian Act) [2, 44–47], has significantly impeded the transmission of parenting practices, values, and beliefs [2]. As noted in our research, the removal of children from indigenous families, including both child apprehension as a consequence for parents who do not wish to attend treatment or not allowing parents to bring children with, minimized access among IPWUID/A who were care providers. These practices contribute to the separation of indigenous families—a central tactic of colonial violence and intergenerational trauma. Our findings emphasize the urgent need for residential substance use treatment programs to explicitly respond to the culturally specific needs of indigenous caregivers that facilitate their capacity to keep their children in their care and in treatment settings.

Connection to indigenous cultures, integration of traditional indigenous medicines, cultural practices within mainstream healthcare services, and meaningful engagement with Indigenous communities are among the many recommendations and strategies that can create cultural safety for indigenous peoples in the healthcare system [13]. Our research builds upon research [40, 48] that provides evidence about the benefits of indigenous cultural interventions that improve overall wellness for IPWUID/A. While the benefits of culturally safe and informed practices for indigenous people has been noted, it remains too often discounted in the delivery of harm reduction services as a viable “treatment” for substance use. Harm reduction approaches, combined with culturally safe and peer-led supports, can increase substance use treatment access for IPWUID/A. Therefore, in order to change how IPWUID/A experience substance use treatment, we need to shift our perceptions on what we consider “recovery” in the context of substance use.

Many participants spoke at great lengths about the significance of peer-led support. Participants favored the support of peer-led substance use programs and considered peer workers to offer more value in their “recovery” than mainstream treatment. Past qualitative research on peer-led programs in relation to substance use, including overdose prevention and response, reveal the many benefits of peer-led models [49–52], such as shared lived experience, nominal power dynamics, and a sense of emotional safety and care that extended beyond the normative support role. Participants’ accounts highlighted how peer involvement facilitated harm reduction practices that promoted a prioritization of the needs of people who use drugs. Significantly, participants noted that indigenous peers are essential to increase success in treatment.

These findings reveal particular implications for IPWUID/A accessing substance use treatment. Our findings suggest that mainstream substance use treatment must expand culturally based, peer-led services for IPWUID/A. It is imperative that the inclusion of these strategies is embedded and informed by the varied experiences of IPWUID/A. Cultural interventions and policies that remove barriers to substance use treatment and that support various modes of “recovery” need to be implemented and evaluated.

## Conclusion

Our study has important limitations. First, our research is limited to those experiences of a unique group of IPWUID/A connected to a community-led organization dedicated to the promotion of harm reduction, and therefore, the extent to which our findings are transferrable to other indigenous peoples warrants further investigation. A further limitation is that our research did not account for the lived experiences of transgender and two-spirit participants and warrants further investigation.

To more effectively meet the needs of IPWUID/A when accessing substance use treatment, it will be necessary to consider the social-structural drivers of violence towards IPWUID/A and how disparities within this population have resulted from the extension of colonialism and racialized power dynamics within mainstream substance use treatment. Our study also reveals how imperative it is that narratives around what we consider “treatment” and “recovery” change to encompass broader notions of harm reduction. Finally, indigenous voices must be acknowledged and addressed by those in the healthcare sector to ensure that substance use treatment is informed by the varied experiences of IPWUID/A in order to deliver culturally safe and responsive peer-led services.

## Data Availability

Primary data for this study is not available as per ethics.

## Abbreviations

IPWUID/A: Indigenous peoples who use illicit drugs and/or alcohol
DTES: Downtown Eastside
WAHRS: Western Aboriginal Harm Reduction Society
BCCSU: British Columbia Center on Substance Use
VANDU: Vancouver Area Network of Drug Users
A.A.: Alcohol anonymous
